# Prevalence and Associated Factors of Acute Traumatic Coagulopathy; a Cross Sectional Study

**Published:** 2017-02-24

**Authors:** Hojjat Derakhshanfar, Ali Vafaei, Ali Tabatabaey, Shamila Noori

**Affiliations:** 1 Emergency Department, Imam Hossein Hospital, Shahid Beheshti University of Medical Sciences, Tehran, Iran.; 2 Emergency Department, Loghman Hakim Hospital, Shahid Beheshti University of Medical Sciences, Tehran, Iran.; 3 Emergency Department, Shahid Beheshti Hospital, Qom University of Medical Sciences, Qom, Iran.; 4 Pathology Department, Imam Hossein Hospital, Shahid Beheshti University of Medical Sciences, Tehran, Iran.

**Keywords:** Blood coagulation disorders, multiple trauma, risk factors, emergency service, hospital, outcome assessment

## Abstract

**Introduction:**

Acute traumatic coagulopathy (ATC) is defined as having evidence of coagulopathy in patients with severe trauma. The aim of this preliminary study was to assess the prevalence and associated factors of ATC in severely traumatic patients presenting to emergency department (ED).

**Methods:**

In this retrospective cross sectional study, all patients with severe traumatic injury and available coagulation profile, presenting to the EDs of two major trauma centers in Tehran, Iran, during one year, were studied. Rate of ATC was determined and the associations with various variables as well as outcome were analyzed using SPSS 21.

**Results:**

246 patients with the mean age of 36.57±17.11 years were included (88.2% male). The mean injury severity score (ISS) was 21.83 ± 7.37 (16 – 54). Patients were resuscitated with 676.83 ± 452.02 (0 – 1500) ml intravenous fluid before arriving at the ED. The maximum and minimum frequencies of ATC were 31.3% based on PTT > 36s and 2.4% based on PT > 18s, respectively. There was a significant association between the occurrence of ATC (PT ratio > 1.2) and ISS > 23 (p = 0.001), abdominal abbreviated injury score (AIS) > 3 (p = 0.003), base deficit > 4 (p = 0.019), pulse rate > 90/minute (p = 0.041), and pH < 7.30 (p = 0.043).

**Conclusion:**

The frequency of ATC in the present series varied from 2.4% to 31.3% based on different ATC definitions. Abdominal AIS > 3 and base deficit > 4 were among the significant independent factors related to ATC occurrence based on stepwise logistic regression analysis.

## Introduction

One third of trauma related deaths are due to hemorrhage, which are generally preventable (1). Acute traumatic coagulopathy (ATC) is defined as having evidence of coagulopathy in patients with severe trauma on admission to emergency department (ED). The term ATC was first introduced in 2003 (2) and recent studies indicated that this abnormality is an endogenous phenomenon, which starts before any intervention (3). This characteristic separates this entity from the better known dilution coagulopathy or consumption coagulopathy in trauma patients. It is estimated that up to 25% of severely injured patients have ATC. These patients have four-fold increased risk of mortality and significantly greater transfusion requirement (4). More importantly, it has been suggested that management strategies targeting ATC may result in significant improvement of outcomes (1). 

Many attempts have been made for diagnosing ATC in the early minutes of admission to ED (1, 5, 6). Unfortunately, currently available laboratory methods do not have the capability of early ATC diagnosis. Therefore, researchers have sought to find clinical factors for early prediction of ATC occurrence, which is vital for initiation of lifesaving treatments (7, 8). Risk stratification of patients based on clinical findings and quick-available laboratory studies could help in early recognition of patients at risk for developing ATC. The aim of this preliminary study was to assess the prevalence and associated factors of ATC in severely traumatic patients admitted to ED. 

## Methods

***Study design and settings:***

In this retrospective cross sectional study, the patients with severe traumatic injury presenting to the EDs of two major trauma centers in Tehran, Iran (Shohadye Tajrish and Imam Hossein Hospitals), during March 2013 to March 2014 were studied. This study was approved by the ethical committee of Shahid Beheshti University of Medical Sciences, and the authors have done the study based on the declaration of Helsinki.

***Participants***

The medical profiles of all patients with available coagulation panel samples, which were admitted to the trauma units of the mentioned hospitals during the study period, were retrospectively evaluated. All patients with severe injury were included. Severe injury was defined as Injury Severity Score (ISS) more than 15 (9). Patients less than 18 years old and subjects with history of bleeding disorder, severe liver dysfunction, and using anticoagulation medications within the last two weeks were excluded. Moreover, in order to exclude patients at risk of dilution coagulopathy, those who had received more than 2 liters of fluids during transport and those who had been referred from other treatment centers were also excluded from the study.

***Data gathering***

Demographic variables (age and gender), trauma mechanism, presenting vital signs (blood pressure, respiratory rate, pulse rate, temperature, O2 saturation), injured body parts, trauma severity based on ISS, laboratory findings (coagulation profile, cell blood count, and blood gas analysis), as well as in hospital outcomes of the patients (mortality, need for ICU admission, need for surgery, and need for blood transfusion) were extracted and collected from the patients’ profile using a predesigned checklist. Coagulation profile consisted of Prothrombin Time (PT), Partial Thromboplastin Time (PTT), PT ratio (patient PT/control PT), and International Normalization Ration (INR). A senior emergency medicine resident, under supervision of an emergency medicine specialist, was responsible for review of patients’ medical profile and data gathering.

***ATC Definition***

Previous studies have used different definitions for ATC. The current available definitions are: PT more than 18s, PTT more than 36s (5), PT ratio greater than 1.2 (1, 10), or International Normalization Ration (INR) greater than 1.5 (7, 8). All four available definitions were used in the present study and the frequency of ATC was individually determined based on each of the mentioned definitions. A combination of the definitions was also considered, i.e. any patient who met any of the criteria was considered to have trauma related coagulopathy. 

***Statistical analysis***

The data were analyzed using SPSS version 21 and presented as mean ± standard deviation (SD) or frequency and percentage. The association between different ATC definitions and measured outcomes were evaluated. Then, the association between ATC occurrence (based on PT ratio > 1.2) and baseline variables was assessed. Continuous variables were compared using Mann-Whitney U test, and categorical data were analyzed using Chi-Square test. After recognition of statically significant variables, clinically relevant cut-points were assigned to these variables to make their use more feasible in the clinical setting. Finally, logistic regression analysis was employed to determine the independent related factors of ATC occurrence.

## Results

***Baseline characteristics***

246 patients with the mean age of 36.57±17.11 (18 – 88) years were included (88.2% male). Table 1 shows the baseline characteristics and outcome of the study population. Motorcycle accidents were the most frequent mechanism of injury (33.7%) and 70.4% of patients had GCS 15 at the time of presenting to ED. The mean severity of injury based on ISS was 21.83 ± 7.37 (16 – 54). Patients were resuscitated with 676.83 ± 452.02 (0 – 1500) ml intravenous fluid before arriving at the ED. The mean duration of ED stay was 9.38 ± 9.83 (0.5 – 67) hours. 

***ATC (frequency and associations)***

Frequency of ATC based on different predefined definitions and its association with measured outcomes is presented in table 2. The maximum and minimum frequencies of ATC were 31.3% based on PTT > 36s and 2.4% based on PT > 18s, respectively. None of the definitions was able to predict in-hospital mortality. 

**Table 1:** Baseline characteristics and outcome of the study population

**Variables**	**Values**	
**Gender**		
Male	216 (88.2)	
Female	29 (11.8)	
**Mechanism of injury**		
Pedestrian	33 (13.4)	
Motorcycle	83 (33.7)	
Car	48 (19.5)	
Rollover	20 (8.1)	
Falling	47 (19.1)	
Assault	10 (4.1)	
Others	5 (2.0)	
**Transport to hospital**		
Ambulance	173 (70.3)	
Unknown	10 (4.1)	
Self-admission	63 (25.6)	
**Glasgow coma scale**		
15	146 (60.6)	
8 - 15	74 (30.7)	
< 8	21 (8.7)	
**Presenting Vital signs**		
Systolic blood pressure (mmHg)	116.45 ± 17.74	
Respiratory rate (/minutes)	17.63 ± 2.78	
Pulse rate (/minutes)	89.46 ± 15.06	
Saturation O2 9%)	94.38 ± 5.33	
Temperature (c)	37.3 ± 0.40	
**Outcome **		
Need for blood products	39 (15.9)	
Need for ICU admission	103 (42)	
Need for surgical intervention	192 (78)	
Mortality	20 (8.1)	

Data were presented as mean ± standard deviation or frequency and percentage.

**Table 2:** Association of acute traumatic coagulopathy occurrence and patients’ outcomes

**Variables**	**Number (%)**	**Outcomes**			
		**Mortality**	**ICU **	**surgery**	**Transfusion**
PT > 18s	6 (2.4)	-	-	-	+
PTT > 36s	77 (31.3)	-	-	+	-
PT ratio > 1.2	28 (11.4)	-	+	-	+
INR > 1.5	27 (11)	-	+	-	+
Combination	94 (38.2)	-	-	+	-

PT: prothrombin time; PTT: partial thromboplastin time; INR: international normalized ratio; ICU: need to admission in intensive care unit;

+: means statistically significant association.

**Table 3:** Association of acute traumatic coagulopathy (ATC) occurrence and different baseline variables

Variable	ATC		P
	**With (n=28)**	**Without (n=218)**	
Male Gender	192 (88)	24 (86)	0.670
Age (year)	32.25 ± 13.74	37.12 ± 17.44	0.221
Transport time (minute)	42.30 ± 39.49	36.34 ± 28.70	0.824
Intravenous fluids (ml)	767.86 ± 419.04	665.14 ± 455.66	0.237
Injury severity score	25.75 ± 9.312	21.33 ± 6.95	0.017
Head and Neck AIS	1.71 ± 1.94	2.09 ± 1.90	0.365
Face AIS	0.57 ± 1.20	0.66 ± 1.13	0.519
Thorax AIS	1.07 ± 1.65	0.98 ± 1.44	0.996
Abdomen AIS	2.07 ± 1.96	1.03 ± 1.48	0.004
Extremities AIS	1.61±1.77	1.56 ± 1.80	0.884
External AIS	0.39 ± 0.86	0.14 ± 0.52	0.083
Presenting vital signs			
Glasgow coma scale	12.75±3.00	13.44±3.01	0.139
SBP (mmHg)	100.32±17.48	117.25±17.66	0.029
Respiratory rate (/minute)	95.07±12.58	88.73±15.23	0.004
Temperature (C)	37.00±0.32	37.04±0.42	0.905
Respiratory rate (/minute)	18.68±4.25	17.49±2.52	0.199
O2 Saturation in the ED	92.70±4.94	94.59±5.36	0.014
Laboratory findings			
Serum pH	7.31±0.13	7.38±0.12	0.044
PaCO2 (mmHg)	40.85±14.62	39.92±11.17	0.788
HCO3	19.91±5.62	23.88±8.67	0.047
Base deficit	5.47±5.61	1.41±6.99	0.026
White blood cell (10^3^/µL)	14.03±5.17	12.15k±4.59	0.034
Platelet count (10^3^/µL)	170.18±88.32	192.53±84.87	0.119
Hemoglobin (mg/dl)	11.51±2.69	12.12±2.79	0.230

Data were presented as mean ± standard deviation or number and percentage. AIS: Abbreviated Injury Score; SBP: systolic blood pressure.

Table 3 summarizes the results of univariate analysis between ATC and different baseline variables. There was a significant correlation between the occurrence of ATC (PT ratio > 1.2) and ISS > 23 (p = 0.001), abdominal AIS > 3 (p = 0.003), base deficit > 4 (p = 0.019), pulse rate > 90 (p = 0.041), and pH < 7.30 (p = 0.043).

Based on the results of a stepwise logistic regression analysis abdominal AIS > 3 (p = 0.001) and base deficit > 4 (p = 0.011) were independent related factors of ATC occurrence. 

## Discussion

The frequency of ATC in the present series varied from 2.4% (PT > 18s) to 31.3% (PTT > 36s) based on different ATC definitions. Abdominal AIS > 3 and base deficit > 4 were among the significant independent related factors of ATC occurrence. 

A high prevalence of ATC has been reported in Uganda with 54% (5), while an Australian study found that only 9.0% of their traumatic patients met the definition of traumatic coagulopathy (8). Two studies from Europe have reported the prevalence of ATC in the range of 24% to 34% (2, 11). 

Most studies have shown a significant association between the presence of ATC and mortality (2, 3, 5, 8, 12). This was not the case in this study. Neither the four definitions used for ATC, nor their combination, were able to predict mortality. We believe this was in part due to the low rate of mortalities in our study, which greatly limited its power to find an association between coagulation tests and mortality. Occurrence of ATC, successfully predicted the necessity for intensive care, and requirement of blood products transfusion. More transfusion and resuscitation requirements are other widely accepted predictable outcomes of ATC (7, 13, 14), yet they are not universal (5). In our study, having ATC would strongly predict higher transfusion requirements.

Genetic factors are well-known contributors to the occurrence of ATC (7, 11); so, the study of this phenomenon in different settings may further expand our understanding of the contributing factors. This has been a longtime interest of researchers since the coagulation profile is rarely made available in the early minutes of ED admission (1). In the absence of a rapid diagnostic tool, management of ATC is currently relying on suboptimal empirical transfusion strategies (14-17). In an attempt to predict the occurrence of ATC before availability of coagulation profile, Mitra et al. introduced the coagulopathy of severe trauma (COAST) score to help in early diagnosis of ATC. The researchers found that scores equal to or greater than 3 are predictive of ATC with appropriate sensitivity and specificity (8). In another study in Germany, Yucel et al. developed a more complicated scoring system aiming to predict ATC and the need for massive transfusion. In the Trauma Associated Severe Hemorrhage (TASH) score system, items such as hemoglobin, base excess, systolic blood pressure, heart rate, confirmed free intra-abdominal fluid, instable pelvic fracture, femur fracture, and male gender are contributing (18). A TASH score greater than 16 has been suggested as the cut-point for prediction of ATC. Other studies have also searched for clinical risk factors of ATC. Abdominal and thoracic trauma, pelvic fractures, mechanism of injury, and evidence of anemia and shock have been suggested as the risk factors for ATC (7, 19). The importance of male gender has also been emphasized (13, 20). In this study, although no association was found between ATC and gender, significant and independent associations were recognized with grade of abdominal trauma, and a base deficit greater than 4. This is in accordance with previous findings, which stated that coagulopathy is more prevalent when severe injury and hypo perfusion are co-existing (21-23). A prospective multi-center cohort study is suggested as the next step for evaluating ATC. 

***Limitations***

This was a preliminary attempt for estimating the prevalence and also recognizing the associated factors of ATC in Iran. The retrograde design of the study predisposes it to several limitations. The most important limitation was incomplete coverage of the target population and having missing data. We selected patients for whom a coagulation panel had been ordered by physicians in the primary order. We believe that since these tests are generally ordered for severely injured patients and those who require operative interventions, we most likely have not missed a large portion of our target population. The low rate of mortality in our studied sample might be owing to selection bias; in which the test results of patients with early mortality might not have been included in their medical profile, leading to elimination from this study. Another more possible explanation for the low reported mortality might be a high incidence of pre-hospital mortality. It has been reported that, in developing countries, more than 70% of deaths due to injuries occur on the scene (21). In such settings most early mortalities never reach hospital-based studies. 

## Conclusion:

The frequency of ATC in the present series varied from 2.4% (PT > 18s) to 31.3% (PTT > 36s) based on different ATC definitions. Abdominal AIS > 3 and base deficit > 4 were among the significant independent factors related to ATC occurrence based on stepwise logistic regression analysis. This reinforces the concept that acute traumatic coagulopathy is triggered when severe injury and hypoperfusion coexist. 
